# Vegans, vegetarians, fish-eaters and meat-eaters in the UK show discrepant environmental impacts

**DOI:** 10.1038/s43016-023-00795-w

**Published:** 2023-07-20

**Authors:** Peter Scarborough, Michael Clark, Linda Cobiac, Keren Papier, Anika Knuppel, John Lynch, Richard Harrington, Tim Key, Marco Springmann

**Affiliations:** 1 Nuffield Department of Primary Care Health Sciences, University of Oxford, Radcliffe Observatory Quarter, Oxford, UK; 2 NIHR Oxford Health Biomedical Research Centre at Oxford, Warneford Hospital, Oxford, UK; 3 Oxford Martin School, University of Oxford, Oxford, UK; 4 Griffith University, Southport, Queensland Australia; 5 Cancer Epidemiology Unit, Nuffield Department of Population Health, University of Oxford, Oxford, UK; 6 Nature-based Solutions Initiative, Department of Biology, University of Oxford, Oxford, UK

**Keywords:** Environmental impact, Environmental impact, Climate-change mitigation, Sustainability

## Abstract

Modelled dietary scenarios often fail to reflect true dietary practice and do not account for variation in the environmental burden of food due to sourcing and production methods. Here we link dietary data from a sample of 55,504 vegans, vegetarians, fish-eaters and meat-eaters with food-level data on greenhouse gas emissions, land use, water use, eutrophication risk and potential biodiversity loss from a review of 570 life-cycle assessments covering more than 38,000 farms in 119 countries. Our results include the variation in food production and sourcing that is observed in the review of life-cycle assessments. All environmental indicators showed a positive association with amounts of animal-based food consumed. Dietary impacts of vegans were 25.1% (95% uncertainty interval, 15.1–37.0%) of high meat-eaters (≥100 g total meat consumed per day) for greenhouse gas emissions, 25.1% (7.1–44.5%) for land use, 46.4% (21.0–81.0%) for water use, 27.0% (19.4–40.4%) for eutrophication and 34.3% (12.0–65.3%) for biodiversity. At least 30% differences were found between low and high meat-eaters for most indicators. Despite substantial variation due to where and how food is produced, the relationship between environmental impact and animal-based food consumption is clear and should prompt the reduction of the latter.

## Abstract (web summary)

Modelled estimates of the environmental impact of dietary choices often fail to reflect true dietary practice. This study links a dietary dataset from 55,000 UK consumers with food-level data on GHG emissions, land use, water use, eutrophication and biodiversity to compare the environmental burden of different levels of meat consumption.

## Main

The substantial impact of the global food system on the environment is well established. It is estimated that the food system was responsible for 18 Gt of carbon dioxide equivalent (CO_2_e) greenhouse gas (GHG) emissions in 2015, comprising 34% of total global GHG emissions that year^1^. The food system is also responsible for 70% of the world’s freshwater use and 78% of freshwater pollution^2,3^. About three quarters of the World’s ice-free land area has been affected by human use, primarily agriculture^4^, and land-use change (primarily deforestation for agriculture) is a major source of biodiversity loss^5,6^.

To feed a growing global population while remaining within proposed safe environmental boundaries for GHG emissions, land use, water use, water pollution and biodiversity loss, we will need changes in diets^7^. Other means to reduce the environmental impact of the food system (for example, technological advances, closing yield gaps, reducing food waste) will not be enough without major dietary change^7,8^. The environmental impact of animal-based foods is generally higher than for plant-based foods because of both direct processes related to livestock management (for example, methane (CH_4_) production by ruminants) and indirect processes through the inefficiency of using crops for animal feed rather than directly for human consumption^3,9,10^. For this reason, proposed diets for global sustainable food production require most high-income countries to radically reduce consumption of animal-based foods and converge on levels that are higher than currently consumed in many low-income countries^8^.

Systematic reviews of modelled dietary scenarios have shown that vegan and vegetarian diets have substantially lower GHG emissions, land use and water use requirements than meat-containing diets^11,12^ and that diets with reduced animal-based foods tend to be healthier and have lower environmental impact^13^. However, modelled dietary scenarios may not reflect true dietary practice, and modelled environmental and health outcomes can be strongly affected by assumptions made by the modellers. Also, previous modelled dietary scenarios have not reflected the considerable variation in environmental indicators due to both region of food production and agricultural production methods^3^ and therefore will have underestimated the uncertainty associated with their findings. While we continue to use average values of environmental impact for food categories, we cannot know whether the observed differences in environmental impact between dietary groups still exist after accounting for variation in food production systems. We therefore need to link data from dietary surveys of real-life dietary patterns with large datasets of environmental indicators to ascertain whether the relationship between animal-based food consumption and environmental outcomes shown in modelling studies is robust.

**Fig. 1:**
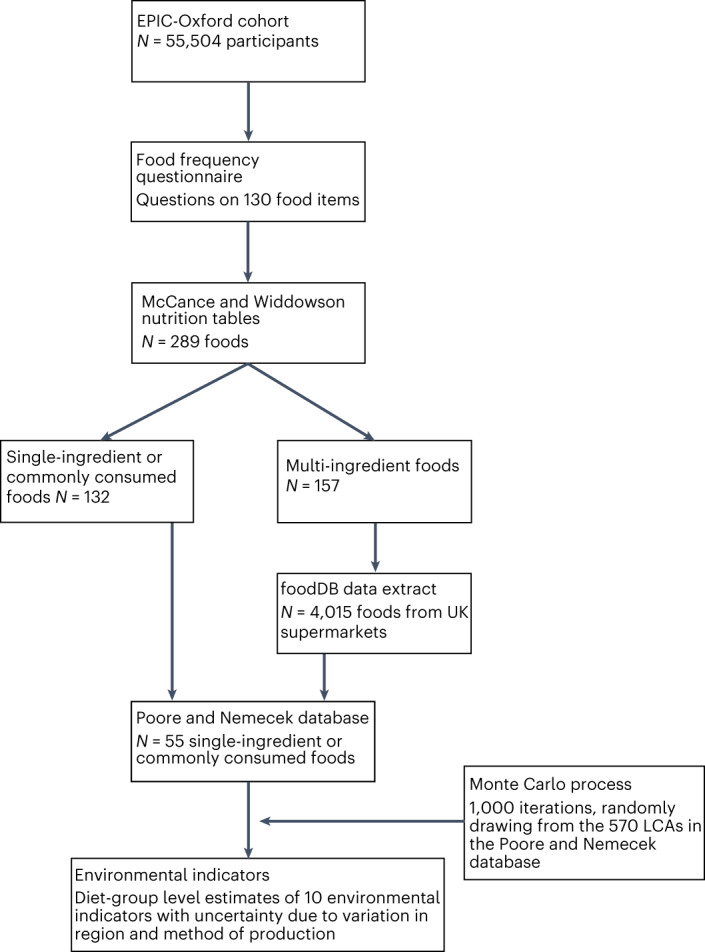
Summary of data linking process. Flow chart shows how data from different sources have been linked for these analyses. Further information about the linkages is provided in the Supplementary Data 1 (Supplementary Section 1).

Previously, we estimated the dietary GHG emissions associated with real-life diet groups in the UK^14^. These estimates only captured one aspect of the environmental impact of food systems, and the data for GHG emissions were derived from a single source with no information about variation within individual food groups due to sourcing or production^15^. Also, GHG emissions data were not presented as disaggregated gases, losing climatically important information^16^. In this paper, we link a validated food frequency questionnaire (FFQ) to estimates from a review of 570 life-cycle assessments^3^ (LCAs) to estimate the GHG emissions (CH_4_, nitrous oxide (N_2_O) and carbon dioxide (CO_2_), in addition to combined CO_2_e emissions), water use, land use, water pollution and biodiversity impact associated with observed diets of vegans, vegetarians, fish-eaters and meat-eaters in the UK (Fig. 1). Our approach allows for direct comparisons of the environmental indicators for each diet group, incorporating uncertainty due to food sourcing and production, and individual-level diet choice.

## Results

The participants and their dietary intake are described in Table 1. Vegans and vegetarians were younger than fish-eaters and meat-eaters, and vegans reported a lower dietary intake of energy than all other diet groups. Fish consumption was similar in fish-eaters and low meat-eaters (with higher levels of consumption in medium and high meat-eaters), suggesting that fish-eaters were not replacing meat with fish. While total dairy consumption was lower in vegetarians and fish-eaters compared to meat-eaters, there was higher consumption of cheese in these two groups.

**Table 1:** Baseline dietary intakes of 55,504 EPIC-Oxford participants overall and by diet group

	Total	Vegans	Vegetarians	Fish-eaters	Low meat-eaters (<50 g d^−1^)	Medium meat-eaters (50–99 g d^−1^)	High meat-eaters (≥100 g d^−1^)
*N*	55,504	2,041	15,751	8,123	9,332	11,971	8,286
Age, mean (s.d.)	44.6 (13.7)	37.3 (13.1)	38.6 (12.7)	41.8 (12.9)	47.5 (13.3)	49.8 (12.6)	49.7 (12.3)
Women (%)	77.2%	63.4%	76.9%	82.2%	80.0%	77.8%	72.1%
Energy intake (kcal d^−1^), mean (s.d.)	1,931 (537)	1,754 (556)	1,879 (530)	1,897 (528)	1,816 (510)	1,940 (502)	2,222 (530)
Grains from bread, cereal, rice and pasta (g d^−1^), mean (s.d.)	213 (103)	251 (117)	235 (103)	231 (105)	204 (103)	189 (95)	190 (94.0)
Potatoes (g d^−1^), mean (s.d.)	82.6 (54.5)	81.6 (67.1)	76.6 (52.1)	72.6 (51.0)	71.6 (49.1)	89.3 (52.2)	107 (58.9)
Beans and pulses (g d^−1^), mean (s.d.)	32.4 (34.3)	60.2 (48.9)	43.6 (40.2)	37.1 (33.4)	25.2 (28.2)	21.6 (24.2)	23.4 (25.2)
Fruit and vegetables (portions per day), mean (s.d.)	6.8 (3.8)	8.7 (5.6)	7.1 (3.9)	7.3 (3.9)	6.9 (4.0)	6.3 (3.2)	6.1 (3.2)
Meat and meat products including poultry (g d^−1^), mean (s.d.)	42.0 (52.9)	0.3 (4.4)^a^	0.4 (5.8)^a^	2.0 (10.7)^a^	28.3 (12.9)	74.0 (14.0)	140 (39.7)
Fish and fish products (g d^−1^), mean (s.d.)	28.4 (31.6)	0.5 (4.6)^a^	0.6 (5.1)^a^	38.9 (33.6)	38.6 (29.5)	43.7 (28.6)	44.2 (29.7)
Cheese (g d^−1^), mean (s.d.)	23.5 (22.1)	1.5 (7.0)^a^	30.0 (25.2)	27.3 (24.0)	22.8 (20.5)	19.8 (18.0)	19.3 (17.1)
Animal milk (ml d^−1^), mean (s.d.)	288 (198)	7.2 (46.1)^a^	260 (203)	273 (190)	300 (186)	331 (182)	349 (187)
Total yogurt (g d^−1^), mean (s.d.)	33.7 (40.8)	2.2 (9.7)^a^	33.7 (40.9)	37.6 (42.5)	38.1 (43.5)	34.4 (39.3)	31.8 (39.2)

*P* value for difference calculated by analysis of variance for all variables except ‘% women’, which is calculated by Pearson’s chi-squared test. The *P* heterogeneity between diet groups was <0.001 for all variables.

^a^Intakes of these foods in these groups are generally nil, but small values are possible as a result of self-assigned diet groups and questionnaire design.

**Fig. 2:**
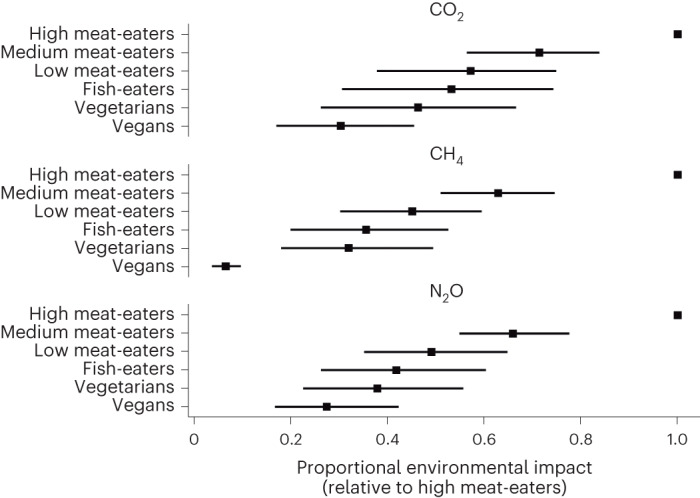
Relative environmental footprint from GHG emissions of diet groups in comparison to high meat-eaters (>100 g d^−1^). Uncertainty intervals are 2.5th to 97.5th percentiles of a Monte Carlo analysis (*n* = 1,000). Source data

**Fig. 3:**
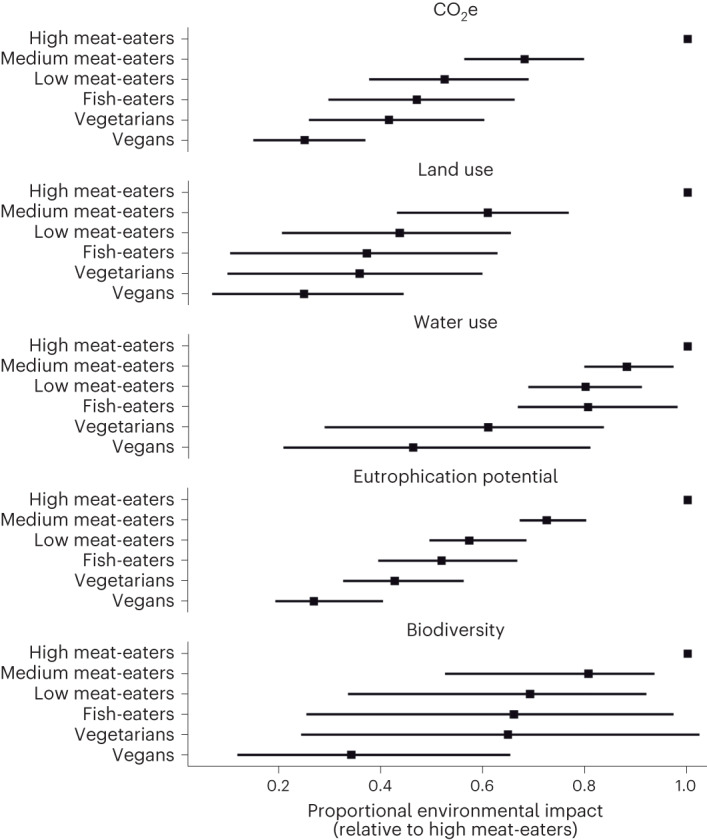
Relative environmental footprint from GWP100, land use, water use, eutrophication potential and biodiversity impact of diet groups in comparison to high meat-eaters (>100 g d^−1^). Uncertainty intervals are 2.5th to 97.5th percentiles of a Monte Carlo analysis (*n* = 1,000). Source data

Estimates of environmental indicators of the diet groups are shown in Tables 2–4, and relative impacts compared to the high meat-eaters are shown in Figs. 2 and 3. The uncertainty associated with sourcing and production is highly correlated between diet groups. This is because food-level draws that produce Monte Carlo iterations with low estimates for the vegan diet group are highly likely to produce low estimates of environmental impact for all other diet groups. For this reason, the results in Tables 2–4 can be used to show uncertainty in absolute estimates of environmental impact for any single diet group, but for comparisons between diet groups the results in Figs. 2 and 3 should be used (which account for the correlation in the uncertainty between diet groups). The results shown in Figs. 2 and 3 represent re-analyses of the dataset and cannot simply be calculated from the data presented in Tables 2–4. Tables with full results for these figures are provided in Supplementary Tables 8–10.

**Table 2:** Dietary GHG emissions (CO_2_, CH_4_ and N_2_O) by diet group, standardized to 2,000 kcal and by age and gender

Diet group	GHG emissions		
	CO_2_ (kg d^−1^)	CH_4_ (g d^−1^)	N_2_O (g d^−1^)
Vegans	2.16 (1.81, 2.94)	4.39 (3.13, 6.37)	0.71 (0.54, 1.01)
Vegetarians	3.33 (2.57, 4.46)	20.21 (15.82, 40.45)	0.98 (0.68, 1.43)
Fish-eaters	3.81 (3.00, 4.92)	22.55 (17.84, 43.63)	1.09 (0.76, 1.52)
Low meat-eaters	4.21 (3.25, 5.40)	28.99 (23.48, 52.41)	1.29 (0.94, 1.76)
Medium meat-eaters	5.34 (3.86, 7.33)	40.88 (32.83, 68.24)	1.73 (1.25, 2.36)
High meat-eaters	7.28 (4.9, 12.23)	65.40 (51.45, 113.89)	2.62 (1.76, 3.90)

Results presented for all adults (*N* = 55,504). All results are presented as median (2.5th percentile, 97.5th percentile) from a Monte Carlo analysis with 1,000 iterations.

**Table 3:** Dietary GHG emissions by diet group aggregated using the GWP100, GTP100 and GWP20, standardized to 2,000 kcal and by age and gender

Diet group	GHG emissions		
	GWP100 CO_2_e (kg d^−1^)	GTP100 CO_2_e (kg d^−1^)	GWP20 CO_2_e (kg d^−1^)
Vegans	2.47 (2.09, 3.36)	2.42 (2.05, 3.29)	2.73 (2.30, 3.64)
Vegetarians	4.16 (3.31, 5.82)	3.84 (3.04, 5.19)	5.35 (4.37, 7.95)
Fish-eaters	4.74 (3.85, 6.27)	4.39 (3.54, 5.72)	6.08 (5.00, 8.73)
Low meat-eaters	5.37 (4.26, 6.99)	4.92 (3.87, 6.31)	7.08 (5.78, 9.93)
Medium meat-eaters	7.04 (5.26, 9.39)	6.34 (4.71, 8.53)	9.55 (7.31, 13.04)
High meat-eaters	10.24 (7.04, 15.95)	8.97 (6.17, 14.15)	14.77 (10.23, 22.55)

Results presented for all adults (*N* = 55,504). All results are presented as median (2.5th percentile, 97.5th percentile) from a Monte Carlo analysis with 1,000 iterations.

**Table 4:** Land use, water use, eutrophication and biodiversity impact by diet group, standardized to 2,000 kcal and by age and gender

Diet group	Land use (m^2^ d^−1^)	Water use (m^3^ d^−1^)	Eutrophication (gPO_4_e d^−1^)	Biodiversity impact (×10^−12^ species extinction per day)
Vegans	4.37 (3.59, 5.90)	0.41 (0.26, 0.77)	10.70 (8.61, 16.28)	1.12 (0.73, 2.55)
Vegetarians	6.01 (5.04, 9.32)	0.53 (0.38, 0.89)	17.27 (14.36, 22.09)	2.08 (1.19, 5.38)
Fish-eaters	6.31 (5.20, 9.68)	0.71 (0.48, 1.63)	21.09 (17.36, 26.52)	2.10 (1.24, 5.51)
Low meat-eaters	8.31 (5.91, 12.95)	0.71 (0.48, 1.70)	23.55 (19.17, 28.88)	2.29 (1.34, 5.90)
Medium meat-eaters	11.28 (7.38, 26.32)	0.78 (0.54, 2.02)	29.61 (23.96, 36.62)	2.77 (1.56, 6.78)
High meat-eaters	16.78 (10.31, 60.84)	0.89 (0.63, 2.04)	40.80 (31.26, 52.27)	3.69 (1.92, 8.92)

Results presented for all adults (*N* = 55,504). All results are presented as median (2.5th percentile, 97.5th percentile) from a Monte Carlo analysis with 1,000 iterations.

For GHG emissions, there was a positive association with amount of animal-based food consumption (Table 2, Fig. 2 and Supplementary Table 8). Dietary CO_2_ emissions for vegans were 30.3% (17.0–45.5%) of the high meat-eaters group. There were also substantial differences in dietary CO_2_ emissions between groups of meat-eaters. Dietary CO_2_ emissions of low meat-eaters were 57.2% (37.8–74.9%) of the high meat-eaters. CH_4_ is a GHG that, in terms of agricultural emissions, is predominantly associated with production of ruminants—it is therefore unsurprising to see wide disparities in CH_4_ emissions associated with the different diet groups. CH_4_ emissions from high meat-eaters were 15.3 (10.3–27.1) times higher than from vegan diets. N_2_O emissions are predominantly associated with fertilizer use, and therefore gradients in N_2_O emissions by diet group are mostly a result of the inefficiencies associated with raising crops for animal feed. This gradient is shallower than for CH_4_ but still substantial, with N_2_O emissions for high meat-eaters 3.6 (2.4–6.0) times higher than for vegans.

Table 3 (and Supplementary Table 9 for relative differences between diet groups) show that using the 100-year Global Temperature change Potential (GTP100) measure resulted in smaller aggregated GHG footprints for all diets, as would be expected given the lower valuation of both N_2_O and especially CH_4_ compared to 100-year Global Warming Potential (GWP100). The ranking of different dietary emission footprints remained the same, but the relative advantage of vegans over diets incorporating animal products decline slightly, with the high meat-eaters responsible for 3.6 (2.4–6.1) times greater GTP100 emissions than vegans, and low meat-eaters 1.8 (1.4–2.6) times greater. For the 20-year Global Warming Potential (GWP20), all footprints were greater, and the relative difference between vegan and other footprints was even more pronounced: high meat-eater diets were 5.1 (3.5–8.4) times greater than vegans.

Table 4, Fig. 3 and Supplementary Table 10 show results for land use, water use, eutrophication and biodiversity impact, all of which show trends in environmental burden from vegans (lowest) to high meat-eaters (highest). For both land use and eutrophication, there is a large difference between the high meat-eaters and all other groups. For eutrophication, the low-meat diet has an impact that is 57.4% (49.6–68.4%) of the high-meat-eating group. For land use, the impact of low meat-eaters is 43.8% (20.7–65.4%) of the high meat-eaters. For both water use and biodiversity impact, there are much bigger gaps for the plant-based groups (for water use, the gap emerges for vegetarians and vegans, whereas for biodiversity impact, it applies to vegans only). However, for both of these environmental indicators, there is far less certainty in both absolute estimates for individual diet groups and also in comparisons between diet groups. Figure 3 shows how this uncertainty propagates, with far wider uncertainty intervals for water use and biodiversity impact than for other measures. For example, the biodiversity impact of vegetarian diets is estimated to be 64.8% of high meat-eaters, but the uncertainty interval (24.5–102.3%) overlap with parity between the groups. The larger uncertainty intervals for these two environmental indicators reflect wide variations in the food-level LCAs.

The results of our sensitivity analyses where we did not standardize diets to 2,000 kcal d^−1^ are shown in Supplementary Section 3 (with equivalent results for the regression-based results in Supplementary Section 2). As shown in Table 1, the measured kilocalorie content of the diet is higher in meat-eaters than in vegetarians and vegans, and high meat-eaters have higher measured kilocalorie intake than low meat-eaters. Therefore, it is unsurprising that not standardizing for kilocalorie intake amplifies the differences in environmental impact across diet groups. In the sensitivity analysis, the environmental footprint of vegan diets is between 5% (CH_4_) and 38% (water use) of the footprint of high meat-eaters. For low meat-eaters, the impact is between 37% (land use) and 67% (water use) of high meat-eaters.

## Discussion

### Statement of principal findings

Diet-related environmental impacts vary substantially by diet groups within this cohort of UK adults which includes a large sample of vegans, vegetarians and fish-eaters. For measures of GHG emissions, land use, water use, eutrophication and biodiversity, the level of impact is strongly associated with the amount of animal-based products that are consumed. Point estimates for vegan diets were associated with less than half of the impact of high-meat-eater (>100 g d^−1^) diets for all indicators, and 95% uncertainty intervals were below 50% for all outcomes except water use and biodiversity. There are also large differences in the environmental impact of diets for groups with lower (but still some) meat consumption. For GHG emissions, eutrophication and land use, the impact for low meat-eaters was at least 30% lower than for high meat-eaters. Large food-level variation in the environmental indicators due to region of origin and method of food production does not obscure differences between diet groups.

### Implications of research

The UK has a legal commitment to a 78% reduction in GHG emissions by 2035 compared to 1990^17^ and of halting biodiversity loss by 2030^18^. The UK Committee on Climate Change has stated that if the government is to achieve its ambitious targets for carbon reductions, then rapid progress must be made across all sectors including implementing measures to encourage consumers to shift diets^19^. Shifts in diets towards plant-based consumption was also emphasized in the 2021 National Food Strategy, which called for a 30% reduction in meat consumption^20^. Previous scenario modelling work has shown that global improvements in food technology, closure of yield gaps and reductions in food waste could potentially reduce dietary GHG emissions by about 15%, primarily through adoption of more efficient technologies in low- and middle-income countries^7^. Our results suggest that much bigger reductions can be achieved by increasing the uptake of plant-based diets, which aligns with other results from this field^7,8,11^.

There are many population-level interventions that could be implemented to support transitions towards lower meat diets. The UK Health Alliance on Climate Change recommends that sustainable diets should be supported by mandatory environmental labelling on foods, regulation of promotions and taxation of high-carbon foods^21^. All of these are variants on policies aimed at increasing healthy diets that either have already been introduced (for example, traffic light labelling, the UK Soft Drink Industry Levy) or have been proposed in the UK Childhood Obesity Plan^22^. The UK Government’s dietary policy is underpinned by its food-based dietary guidelines (FBDGs), known as the Eatwell Guide^23^. A recent systematic review of national FBDGs found that the large majority are not compatible with the proposed downscaling of ‘planetary boundaries’ for food production—if the UK population consumed the diet recommended by the Eatwell Guide, it would not stay within boundaries for GHG emissions, water use, land use and eutrophication suggested by the paper^24^. Incorporating environmental sustainability into FBDGs (such as the Eatwell Guide proposed by Plant-based Health Professionals UK^25^) may be the first step towards implementation of population-level policies that have been shown to support shifts away from animal-based foods^26^.

### Strengths and limitations

This paper uses one of the largest datasets available on the diets of vegans and vegetarians to compare the environmental impact of different diet groups over ten environmental measures. The analyses contribute to the literature that shows the benefit of low-meat diets for reduction of GHG emissions^14^, land use, water use, water pollution and biodiversity. The paper uses only empirical measures of diet, thereby verifying previous modelling work that has suggested multiple environmental benefits of low-meat diets^7,8,27^. By using self-identification as vegan, vegetarian and fish-eater, we ensure that our methods include all dietary patterns within those categories including those that breach some of the definitions of the groups—this means our estimates are likely to reflect real dietary practices as opposed to comparison of idealized diet groups.

A key strength of our analysis is that it incorporates the uncertainty around the environmental parameters drawn from a review of 570 LCAs covering results from over 38,000 farms in 119 countries covering five continents^3^—henceforth, ‘the Poore and Nemecek database’. Doing this shows that although uncertainty for any single food group is large, when this uncertainty is combined over multiple food groups to produce aggregated dietary estimates, we can still observe clear differences between diet groups. Our primary results are based on a Monte Carlo analysis where 1,000 estimates of each food’s environmental impact are produced based on varying measures due to food sourcing and production methods. In our secondary results (shown in Supplementary Tables 1 and 2 and based on regression models that take the median estimate of the environmental parameter for each food group and ignore the underlying variation), not only are the confidence intervals much tighter than in the primary analysis, but the point estimates are also lower. The discrepancy between the two sets of results is due to the computational mathematics involved with combining multiple distributions, many of which are heavily right-skewed, all of which are bounded by zero, and in which negative scalars are not possible (as negative consumption of food is not possible). Although each random draw from the food group distributions is equally likely to be either lower or higher than the median, draws that are higher than the median are, on average, further from the median than those that are lower. When summed, these random draws produce median estimates that are larger than the sum of the medians for the individual food groups. The same principle is shown by rolling two dice. For two normal 1–6 dice (which have no skew), the median score when rolling two dice is 7, which is twice the median score for rolling each dice separately (3½). However, consider rolling two ‘doubling dice’ from backgammon that are heavily right-skewed (with faces 2, 4, 8, 16, 32 and 64). Here, the median score when rolling two dice is 35, much higher than the sum of the median scores for each single dice (which is 12).

Our secondary results (shown in the Supplementary Information) show that ignoring the uncertainty around food-level parameters can result in both underestimation of the uncertainty in diet-level outcomes and bias in the results which can reduce observed differences between diet groups. For example, our primary results show a difference in water use between high meat-eaters and vegans of 480 l d^−1^, with high meat-eaters consuming 2.2 times as much water as vegans, whereas the secondary results show an absolute difference of 210 l d^−1^ and a relative difference of 1.7. The issue of food-level uncertainty affects all areas of nutritional epidemiology that rely on food diaries or FFQs to estimate dietary intake. For example, estimates of sugar consumption produced by these methods do not account for uncertainty in the sugar level of food groups, but we know that wide variability in sugar levels for similar foods exists^28^.

An additional contribution of our research was providing disaggregated GHG emissions and exploring multiple CO_2_-equivalence metrics, whereas most previous studies report only GWP100 CO_2_e. Reporting emissions only as aggregated GWP100 totals results in ambiguity in climate impacts^29^, whereas providing footprints under multiple metrics gives users insight into temporal differences where there are both short- and long-lived gases involved, as highlighted by the Life Cycle Initiative^30^. As food system emissions contain important amounts of CH_4_, a relatively short-lived gas, metric selection can have a pronounced impact on CO_2_e emission reporting^31^. Here, however, using the alternative pulse-emission metrics explored in this study did not greatly affect results, with a fairly small change in total footprints and relative performance between dietary groups. A caveat is that emissions data from the Poore and Nemecek database are not separated into different gases, and while they are categorized to broadly infer gas compositions (for example, assuming that the CO_2_e emissions reported for fertilizer application represented N_2_O, and enteric fermentation CO_2_e represented CH_4_), for other components we had to assume emissions were entirely CO_2_. We reiterate calls for studies on GHG emissions, particularly those relating to agriculture and food, to provide disaggregated emissions to enable the most reliable analyses^31^.

Our analyses are subject to the following further limitations. The data on the environmental footprint of foods are taken from a snapshot of food and drink on sale in the UK in 2019 linked to the most comprehensive publicly available dataset of LCAs of the environmental impact of foods currently available^3^. However, the data on dietary consumption were collected in the 1990s, and dietary preferences are likely to have changed since then. This is mitigated somewhat by the fact that the FFQ was linked to the environmental footprint of food and drink on sale in the UK in 2019, but this will not account for category-level changes in consumption since the 1990s. More recent datasets of dietary consumption in the UK are available, including datasets based on a representative sample of the UK population (for example, Kantar Fast-Moving Consumer Goods panel^32^ and the National Diet and Nutrition Survey^33^). However, the European Prospective Investigation into Cancer and Nutrition (EPIC)-Oxford dataset (used for this analysis) is the most recent data available in the UK that has a large sample of vegan and vegetarian diets, necessary for these analyses. Data collection is underway on the Feeding the Future study^34^, which aims to update estimates of food intake in vegans and vegetarians (and meat-eaters) in the UK. Updating our analyses using more timely data will provide evidence of whether trends in new meat and dairy alternatives have affected the environmental impact of plant-based diets.

Our database of food and drink on sale in 2019 was not adjusted for sales, so we were not able to put extra weight on more popularly consumed foods. For our analyses, we standardized daily diets to 2,000 kcal so that differences between diet groups are entirely a result of the composition of the diets—this may result in underestimates of the difference between diet groups as meat-eaters tend to consume more calories than vegans and vegetarians^35^. Our sensitivity analysis (Supplementary Tables 5–7 and 11–13) shows results that have not been standardized for energy content, which suggests larger differences between the diet groups, but these figures should be treated with caution as some of the difference in kilocalorie intake between groups is caused by artefact. For example, the FFQ used to estimate dietary consumption assumes fixed portion sizes for food groups, but it is likely that portion sizes of cereals, fruit and vegetables are higher in those consuming more plant-based diets.

The FFQ that we used has been validated against food records and biomarkers for estimation of the nutritional quality of the diet, but no such validation has taken place for estimating environmental outcomes. However, a previous validation study compared dietary GHG emissions estimated by a different FFQ with estimates from a 24 h diet recall and showed acceptable levels of agreement between the two^36^. The FFQ in our study did not measure agricultural production methods, so differences between diet groups based on (for example) differing levels of consumption of organic produce could not be assessed. While we included multiple environmental indicators in our analyses, there are other ethical aspects that vary by region and method of agricultural production that are not included here (for example, agricultural working conditions, animal welfare). Finally, as the Poore and Nemecek database is not comprehensive and our uncertainty analyses are not weighted towards more common food production practices, our uncertainty intervals do not fully incorporate all the uncertainty associated with these comparisons between diet groups. As new agricultural practices aimed at reducing the environmental impact of the food system (for example, feed additives, genetic selection, lab-grown meat) becomes more widespread and LCA data become more readily available, our analyses should be updated.

### Comparison with other literature

By scaling our results to the national level, we can compare our absolute estimates of environmental impact with other estimates from the literature. To do this, we used data from the UK’s gold standard diet monitoring programme, the National Diet and Nutrition Survey^33^, which estimated that in 2016–2019 the average consumption of all meat (that is, processed and unprocessed meat including poultry but excluding fish) in 19–64 year olds was 99 g d^−1^, and 77 g d^−1^ in the 65+ age group. We estimated the prevalence of vegans and vegetarians using data from a recent Ipsos Mori survey^37^. Using these data to scale our results to the population of the UK, we estimate that the annual dietary environmental footprint of adults in the UK amounts to 120 MT of CO_2_e, 230,000 km^2^ of agricultural land, 15 km^3^ of agricultural water, 690 kT of phosphate equivalents (PO_4_e) and 0.06 terrestrial vertebrate species destined for extinction. Our estimate of 120 MT of CO_2_e is similar to the most recent estimate from EDGAR-FOOD (Emissions Database for Global Atmospheric Research)^38^, which produces globally comparable estimates using Food and Agriculture Organization of the United Nations food balance sheet data and estimates UK food systems emissions in 2015 to be 113 MT of CO_2_e. Our estimates for water use, eutrophication and biodiversity are similar to results for the UK published by the World Wildlife Fund^39^ of 19 km^3^ of agricultural water, 645 kT of PO_4_e and 0.03 species destined for extinction each year. While our estimate of total GHG emissions is similar to that from EDGAR-FOOD, the proportion of individual gases is different. For our estimates, the contribution to CO_2_e of N_2_O is about 7% for all diet groups, and for CH_4_ the contribution increases from 6% in vegans to 21% in high meat-eaters. Equivalent estimates from EDGAR-FOOD for the UK are 17% for N_2_O and 35% for CH_4_. This may be a result of discrepancies in how we derive separate N_2_O, CH_4_ and CO_2_ emissions making inferences from the Poore and Nemecek database, as noted above, and the way separate gases are handled in the Food and Agriculture Organization Statistics Division (FAOSTAT) emissions in EDGAR-FOOD, further highlighting the challenges in obtaining individual gas data.

Previous estimates of dietary GHG emissions for vegans, vegetarians, fish-eaters and meat-eaters in the EPIC-Oxford cohort have been made using a similar method based on GHG emissions data from a single study^14^. The estimates presented here are slightly lower for plant-based diet groups and slightly higher for meat-eating groups. Other studies have compared the environmental impacts of observed diet groups defined by exclusion of meat or dairy^40–42^, but they have not included as many environmental measures as here nor incorporated uncertainty in estimates due to region of origin and production method. Dietary GHG emissions for US vegetarians in the Adventist Health Study 2 cohort^41,42^, standardized to a 2,000 kcal diet, were 70.8% (70.5–71.2%) of emissions from non-vegetarian diets, similar to the difference between vegetarians and the medium meat-eaters (58.5%) observed in our sample. An analysis of 29,210 French adults in the NutriNet-Sante Study included data on 464 pesco-vegetarians (equivalent to fish-eaters in our study), 406 vegetarians and 297 vegans^40^. For both GHG emissions and land use, that study^40^ found the same relationship as shown in our paper, with lowest environmental impact for vegans, similar impact for vegetarians and fish-eaters, and highest impact for meat-eaters. They also found similar relative differences between vegans and meat-eaters, with dietary GHG emissions of vegans being 24.5% (19.2–29.8%) of the meat-eaters and 35.6% (29.9–41.3%) for land use.

## Conclusion

There is a strong relationship between the amount of animal-based foods in a diet and its environmental impact, including GHG emissions, land use, water use, eutrophication and biodiversity. Dietary shifts away from animal-based foods can make a substantial contribution to reduction of the UK environmental footprint. Uncertainty due to region of origin and methods of food production do not obscure these differences between diet groups and should not be a barrier to policy action aimed at reducing animal-based food consumption.

## Methods

See the data availability statement for details of where to access the data for this study.

### Recruitment and dietary assessment

Data on food consumption comes from the baseline data collection of the EPIC-Oxford prospective cohort study^43^. Between 1993 and 1999, data were collected on 65,411 adults aged 20 years and over. Individuals were recruited through advertising in vegetarian and health food magazines, through direct mailout from vegetarian and vegan societies and through collaborating general practices. Recruited individuals were then encouraged to recruit acquaintances. All participants were residents in the UK.

Dietary assessment was conducted using a 130-item FFQ that assesses the usual levels of consumption of food items over the previous 12 months. The FFQ has been validated against weighed food records and several recovery and concentration biomarkers^44^. The FFQ was used to estimate food group and nutrient intakes, and participants were classified into self-identified dietary groups (vegans, vegetarians, fish-eaters and meat-eaters) by their responses to the following four yes or no questions:

- Do you eat any meat (including bacon, ham, poultry, game, meat pies, sausages)? (Vegans, vegetarians and fish-eaters respond ‘No’.)
- Do you eat any fish? (Vegans and vegetarians respond ‘No’.)
- Do you eat any eggs (including eggs in cakes or other baked goods)? (Vegans respond ‘No’.)
- Do you eat any dairy products (including milk, cheese, butter, yoghurt)? (Vegans respond ‘No’.)

In addition, we split the meat-eaters into three groups based on amount of daily consumption: low meat-eaters (0 to <50 g d^−1^), medium meat-eaters (≥50 to <100 g d^−1^) and high meat-eaters (≥100 g d^−1^). These cut-offs were selected as they split the cohort into three similarly sized groups and allow for direct comparison with other published studies.

For these analyses, we excluded participants if they were aged 80 years or over, or under the age of 20 years at recruitment, did not complete at least 80% of the FFQ, did not complete the questions required for classification into dietary groups, or produced estimates of daily energy intake that were deemed unfeasible^45^ (for men, <3.3 MJ or >16.7 MJ, and for women <2.1 MJ or >14.7 MJ; *n*_total excluded_ = 9,907).

### Environmental data

The environmental data on emissions of CH_4_, N_2_O and CO_2_ and estimates of water use, land use, eutrophication (dense growth of algae and plant life caused by excess nitrogen and phosphorus levels in the water) and biodiversity impact on terrestrial vertebrates, were taken from the Poore and Nemecek database—a review of 570 LCAs covering results from over 38,000 farms in 119 countries covering five continents^3^. Disaggregated GHG estimates were not always available in the Poore and Nemecek database. Where they were not available, CH_4_ and N_2_O emissions were assumed to be the sum of emissions from agricultural practices where these GHGs dominate (for example, CH_4_ for enteric fermentation) and CO_2_ was assumed to be the remaining component. We selected all of the environmental indicators available in the Poore and Nemecek database except acidification (because of gaps in the data) and water scarcity (because it is heavily based on water use, which we already use). The life cycle estimates are valid up to the retail setting. The database contains LCAs published between 2000 and 2016 that met inclusion criteria based around a minimum standard of reporting.

We used data on the GHGs to estimate aggregated GWP100 (CH_4_ conversion factor = 27, N_2_O = 273) using conversion factors from the Sixth Assessment Report of the Intergovernmental Panel on Climate Change^46^. As agricultural emissions contain a substantial non-CO_2_ component, aggregated CO_2_e emission footprints can vary depending on the method used to define CO_2_-equivalence. Following United Nations Environment Programme and Society of Environmental Toxicology and Chemistry (UNEP-SETAC) Life Cycle Initiative guidance^30^, we explored total dietary footprints using two additional metrics in addition to the *de facto* standard GWP100. These were the GTP100 (CH_4_ conversion factor = 11, N_2_O = 297), suggested as representing longer-term climate impacts, and the GWP20 (CH_4_ conversion factor = 86, N_2_O = 268), suggested as providing insight into very short-term impacts.

The exact measures used for our environmental measures are:

- GHG emissions measured in kg total GWP100/GTP100/GWP20 CO_2_e, kgCO_2_e, and separate emissions of CH_4_ and N_2_O in grams, and CO_2_ in kilograms.
- Agricultural land use, including both cropland and pastureland, measured in m^2^.
- Agricultural water use, measured in m^3^ (1 m^3^ = 1,000 litres).
- Eutrophication potential measured in g of PO_4_e, gPO_4_e (combining the eutrophication potential of major nitrogen and phosphorus pollutants).
- Biodiversity impact, which is measured as the number of species destined for extinction as a result of agricultural practices. This variable accounts for the impacts of land cover expansion (for example, conversion of natural ecosystems to cropland or pastureland) and ongoing use of agricultural land, and is weighted depending on the location of land use^47^. The index is specific to 170 crops in 184 countries^48^. The measure we use only accounts for the impact of land-based food production on terrestrial vertebrates, and therefore does not account for biodiversity loss of terrestrial plants or invertebrates, or any aspect of marine biodiversity. This measure is not usually used to assess the potential biodiversity impact of diets consumed by a single individual on a single day. Therefore the units of measurement are very small (10^−12^ species destined for extinction), and the measure is better understood as a comparative measure across diet groups.

### Linkage of datasets

The process for linking EPIC-Oxford data with environmental assessments is summarized in Fig. 1, and tables demonstrating the links at each stage in the process are provided in the Supplementary Data 1 (Supplementary Section 1). We first ascertained the relevant food codes corresponding to the 130-item FFQ using the UK foods composition tables available at the time of data collection; this yielded 289 foods codes^49,50^. We then linked the 289 food items with the environmental indicators data via an intermediary step involving a database (foodDB) of all food and drink items available for purchase in eight UK online supermarkets^28^. We linked an extract of 57,000 food and drinks from October 2019 with the environmental dataset using a process that is described in detail elsewhere^51^. Briefly, each ingredient in each product in the data extract was linked with food categories from the Poore and Nemecek database. Then, for each food product, the percentage composition of each ingredient was estimated in a two-stage process: first, the per cent composition provided in the ingredients list by the manufacturer was used if it was provided; second, for the remaining ingredients, we used an algorithm to estimate the per cent composition of remaining ingredients using composition and nutrition information from similar products and following UK food-labelling regulations, such that the composition of all ingredients in a product sums to 100% and each ingredient accounts for at least as much of the product as the subsequent ingredient. The accuracy of the approach was assessed by applying the algorithm to a subset of 1,550 foods in the database where the percentages of all ingredients were known. In the extreme scenario where it was assumed that no per cent composition of any ingredient was known, the algorithm on average produced estimates of environmental measures that were within 2% of the known environmental measure across all assessed products. While most products and ingredients identified in foodDB do not provide information on the agricultural methods used for their production, where we identified foods or ingredients labelled as ‘organic’ we linked them with data on LCAs for organic production methods in the Poore and Nemecek database.

To link the 289 food items from the FFQ with environmental data, we first identified those foods (*n* = 132) that could be linked directly with data from the Poore and Nemecek database. These foods were either single-ingredient foods (for example, peaches, salmon, beefsteak, milk) or were commonly consumed staples (for example, bread, alcoholic drinks). These links are shown in the Supplementary Data 1 (Supplementary Section 1).

For the remaining 157 foods, we matched on keywords with products from the food and drinks in the foodDB data extract. We matched with 4,015 unique food and drink products. The median number of product matches was 11, ranging from 1 for frozen mousse to 500 for chips. To link with multiple foods, we used the mean of the environmental impact. These links are shown in the Supplementary Data 1 (Supplementary Section 1), as are the links between these 4,015 food and drink products and the food categories from the Poore and Nemecek database. We made adjustments to convert from weight as sold to weight as consumed using conversion factors from our previous study^14^.

### Statistical analysis

We compared age, gender and measures of dietary intake across the diet groups, and differences were assessed by analysis of variance for continuous variables and Pearson’s chi-squared test for categorical variables. To account for different energy intakes across diet groups, environmental measures were standardized to a daily diet of 2,000 kcal by proportionately scaling all consumption of different food and drinks. This allowed us to isolate the differences between the diet groups that are purely a result of the composition (rather than the amount) of food consumed. As kilocalorie intake varies by age and gender, and these variables also vary by diet group, standardizing the kilocalorie intake also protects our results from confounding. In addition, standardizing the kilocalorie intake of diets avoids the potential for artificial differences that could result if the average portion sizes for fruit, vegetables and cereals differ across diet groups. However, standardizing by kilocalorie intake also obscures differences that result from variation in kilocalorie intake by diet group; therefore, as a sensitivity analysis we reproduced all results without standardization to a daily diet of 2,000 kcal.

All of the results that compare environmental measures by diet group have been standardized by the age and gender breakdown in the full EPIC-Oxford sample, so that the influence of age and gender are removed from comparisons. Our results are presented for both genders combined. We have also analysed the data separately for men and women and did not find any differences for any environmental indicators in our primary analyses.

Our primary results are derived from a two-stage Monte Carlo analysis that accounted for uncertainty due to variation in agricultural production methods and where food is produced. For example, the EPIC-Oxford FFQ collects data on consumption of beef. This FFQ item is linked with two items from the McCance and Widdowson nutrition tables (beefsteak and beef fat). The environmental footprint of beefsteak varies depending on how it is produced (for example, pasture fed or intensively reared) and where it is produced (for example, UK or Brazil). This variability is captured by the LCAs in the Poore and Nemecek database—there are 24 LCAs of ‘bovine meat (beef herd)’ in the database. Stage 1 of our Monte Carlo analysis produced distributions of environmental indicators for all of the foods that were linked to the EPIC-Oxford FFQ simultaneously. For each food, we randomly drew 1,000 samples from the distributions of each environmental indicator in the Poore and Nemecek database (for multi-ingredient foods, this would involve drawing across multiple Poore and Nemcek categories—see the Supplementary Data 1 for more information). In stage 2, we used these 1,000 estimates of food-level environmental indicators to generate 1,000 estimates of the environmental indicators for the diets of each of the EPIC-Oxford participants. The 95% uncertainty intervals around our primary results are taken from the 2.5th and 97.5th percentiles of these iterations. Ratios of the environmental impact are presented, with high meat-eaters as the baseline group. These ratios (and accompanying 95% uncertainty intervals) are the median (and 2.5th and 97.5th percentiles) from results derived separately in each of the 1,000 iterations.

Our secondary analysis accounts for uncertainty due to variation in individual-level diet choices for the EPIC-Oxford participants. We estimated marginal results from a regression analysis adjusted for age and gender, where environmental indicators are fixed at the median level from the Poore and Nemecek database. The marginal results are equivalent to the age and gender standardized results from the primary analysis but only incorporate uncertainty from sampling variance. The secondary results are shown in Supplementary Tables 1 and 2.

## Supplementary information

Supplementary Information Supplementary Tables 1–13.

Supplementary Data 1 Supplementary data tables.

## Data Availability

Data on food consumption comes from the EPIC-Oxford study: the data access policy for the EPIC-Oxford study is available at the study website (www.epic-oxford.org/data-access-sharing-and-collaboration/). Data on the environmental footprint of 57,000 foods from the foodDB project are available from the Oxford Research Archive (https://ora.ox.ac.uk/objects/uuid:4ad0b594-3e81-4e61-aefc-5d869c799a87). Data on environmental LCAs are part of the HESTIA project, which can be accessed at https://www.hestia.earth/. Source data are provided with this paper.

## Source data

Source Data Fig. 2 Source data.
Source Data Fig. 3 Source data.

## Code availability

Code for this project can be found at https://github.com/PeteScarbs/environment-impact-of-diets.
