# Supplementary material for: Vegans, vegetarians, fish-eaters and meat-eaters in the UK show discrepant environmental impacts
Source: Nat Food. 2023 Jul 20;4(7):565–74. doi: 10.1038/s43016-023-00795-w (PMC10365988; doi:10.1038/s43016-023-00795-w)
Supplement: Supplementary file 1 — Supplementary Tables 1–13. [file 43016_2023_795_MOESM1_ESM.pdf]

nature food

Article

https://doi.org/10.1038/s43016-023-00795-w

Vegans, vegetarians, fish-eaters and meat-eaters in the UK show discrepant environmental impacts

In the format provided by the authors and unedited

Vegans, vegetarians, fish-eaters and meat-eaters in the UK show discrepant environmental impacts

Peter Scarborough, Michael Clark, Linda Cobiac, Keren Papier, Anika Knuppel, John Lynch, Richard Harrington, Tim Key, Marco Springmann

S2: Secondary results from regression-based analyses

The results shown in this appendix are based on marginal outcomes of regression models with the environmental variables as the outcome and predictor variables of age (categorical by ten year groups), gender and diet group. For supplementary tables 1 and 2, all data were standardised to 2000kcal before inclusion in the regression models. The results in these tables are directly comparable with the analyses reported in the main paper. For supplementary tables 3 and 4, the data were not standardised to 2000kcal – the results in these tables are comparable with our sensitivity analysis where we did not adjust for kcal consumption (results of the sensitivity analysis are shown in supplementary material S3). Point estimates produced by the marginal outcomes are equivalent to mean estimates for the diet group after direct standardisation to the age and gender profile of the EPIC-Oxford sample. This is exactly equivalent to the method applied in the Monte Carlo analyses used for the primary outcome. The difference in the results of the primary and secondary analyses is therefore entirely due to the incorporation of uncertainty due to sourcing and production of foods, which is included in the primary outcomes and excluded in the secondary outcomes reported here. The 95% confidence intervals produced in these secondary analyses is due to sampling uncertainty, which incorporates variation due to individual-level dietary choice.

Supplementary Table 1 Dietary greenhouse gas emissions by diet group, standardised to 2000kcal and by age and gender. Results from marginal analysis of regression models, and presented for all adults (n = 55,504).

	Greenhouse gas emissions, mean (95% uncertainty intervals)		
Diet group	*GWP100 CO_2e (kg/d)*	*CH_4 (g/d)*	*N_2O (g/d)*
Vegans	2.56 (2.49, 2.64)	4.32 (3.78, 4.86)	0.59 (0.57, 0.61)
Vegetarians	3.70 (3.67, 3.73)	20.62 (20.43, 20.82)	0.60 (0.59, 0.60)
Fish-eaters	4.11 (4.08, 4.15)	21.09 (20.82, 21.35)	0.66 (0.66, 0.67)
Low meat-eaters	5.08 (5.05, 5.11)	29.03 (28.78, 29.28)	0.90 (0.90, 0.91)
Medium meat-eaters	6.54 (6.51, 6.57)	38.62 (38.40, 38.84)	1.28 (1.27, 1.29)
High meat-eaters	8.98 (8.94, 9.02)	54.59 (54.33, 54.86)	1.93 (1.93, 1.94)

Supplementary Table 2 Land use, water use, water pollution and biodiversity impact by diet group, standardised to 2000kcal and by age and gender. Results from marginal analysis of regression models, and presented for all adults (n = 55,504)

Diet group	*Land use (m^2/d)*	*Water use (000s L/d)*	*Eutrophication (gPO_4e/d)*	*Biodiversity impact (10^{-12} species extinction/d)*
Vegans	4.23 (4.10, 4.35)	0.31 (0.30, 0.31)	10.59 (10.35, 10.83)	0.81 (0.79, 0.82)
Vegetarians	5.87 (5.82, 5.91)	0.38 (0.38, 0.39)	16.88 (16.79, 16.97)	1.32 (1.31, 1.32)
Fish-eaters	5.76 (5.70, 5.83)	0.46 (0.46, 0.46)	19.91 (19.79, 20.03)	1.30 (1.30, 1.31)
Low meat-eaters	7.19 (7.13, 7.25)	0.49 (0.49, 0.49)	22.73 (22.62, 22.84)	1.44 (1.43, 1.45)
Medium meat-eaters	9.60 (9.54, 9.65)	0.52 (0.51, 0.52)	26.76 (26.66, 26.86)	1.60 (1.59, 1.60)
High meat-eaters	14.05 (13.98, 14.11)	0.52 (0.52, 0.53)	32.29 (32.17, 32.41)	1.83 (1.82, 1.84)

Supplementary Table 3 Dietary greenhouse gas emissions by diet group, standardised by age and gender *but not standardised by kcal consumption*. Results from marginal analysis of regression models, and presented for all adults (n = 55,504). (SENSITIVITY ANALYSIS)

	Greenhouse gas emissions, mean (95% uncertainty intervals)		
Diet group	*GWP100 CO_2e (kg/d)*	*CH_4 (g/d)*	*N_2O (g/d)*
Vegans	2.12 (2.04,2.19)	3.24 (2.65, 3.82)	0.50 (0.47, 0.50)
Vegetarians	3.42 (3.39,3.45)	19.12 (18.82, 19.41)	0.54 (0.54, 0.57)
Fish-eaters	3.87 (3.84,3.91)	20.00 (19.71, 20.29)	0.64 (0.64, 0.64)
Low meat-eaters	4.49 (4.46,4.53)	25.59 (25.29, 25.88)	0.81 (0.81, 0.81)
Medium meat-eaters	6.15 (6.11,6.18)	36.18 (35.88, 36.47)	1.21 (1.21, 1.21)
High meat-eaters	9.66 (9.62,9.70)	58.82 (58.53, 59.12)	2.08 (2.08, 2.08)

Supplementary Table 4 Land use, water use, water pollution and biodiversity impact by diet group, standardised by age and gender *but not standardised by kcal consumption*. Results from marginal analysis of regression models, and presented for all adults (n = 55,504). (SENSITIVITY ANALYSIS)

Diet group	*Land use (m^2/d)*	*Water use (000s L/d)*	*Eutrophication (gPO_4e/d)*	*Biodiversity impact (10^{-12} species extinction/d)*
Vegans	3.52 (3.39,3.66)	0.26 (0.25,0.26)	8.76 (8.49,9.04)	0.68 (0.66,0.70)
Vegetarians	5.45 (5.40,5.50)	0.36 (0.35,0.36)	15.63 (15.53,15.73)	1.22 (1.22,1.23)
Fish-eaters	5.46 (5.40,5.53)	0.43 (0.43,0.44)	18.74 (18.61,18.88)	1.23 (1.22,1.24)
Low meat-eaters	6.39 (6.33,6.45)	0.44 (0.44,0.44)	20.22 (20.09,20.34)	1.29 (1.28,1.30)
Medium meat-eaters	9.03 (8.97,9.08)	0.49 (0.49,0.49)	25.30 (25.19,25.42)	1.53 (1.52,1.54)
High meat-eaters	15.12 (15.06,15.19)	0.57 (0.57,0.57)	34.91 (34.78,35.05)	1.99 (1.98,2.00)

S3: Sensitivity analysis with results not standardised to 2000kcal and full results of relative environmental footprints (as displayed in figures 2 and 3)

All of the primary analyses for this paper adjust results to 2000kcal for daily intake of all diet groups (vegans, vegetarians, fish-eaters and meat-eaters). There are a number of reasons for this standardisation which are outlined in the main paper. As a sensitivity analysis, we estimated the environmental impact of the diet groups *not* standardised to 2000kcal and we present the results here. Since mean energy intake of vegans and vegetarians is lower than for meat-eaters (see Table 1), not standardising for energy intake amplifies the differences in environmental impact of the food groups. The results for absolute environmental impact are displayed in supplementary tables 5, 6 and 7.

Results in supplementary tables 8, 9 and 10 show, for each environmental measure, the ratio of each diet group compared with the high meat-eaters group from the primary analysis. We calculated the ratios separately for each of the 1000 Monte Carlo iterations that were used for our primary analyses. We then calculated the median, 2.5th percentile and 97.5th percentile of the ratios to display in these tables and in figures 2 and 3. The median, 2.5th percentile and 97.5th percentile of

the ratios may not be taken from the same draws as the main results shown in Tables 2-4, which explains why the results reported here are not identical to crude ratios that can be estimated from the results in this paper.

Results in supplementary tables 11, 12 and 13 are equivalent results for the ratio of each diet group compared with the high meat-eaters group, but this time for the sensitivity analysis where results are not standardised to 2000kcal.

Supplementary Table 5 Dietary greenhouse gas emissions (CO2, CH4 and N2O) by diet group standardised by age and gender *but not standardised by kcal consumption*. Results presented for all adults (n = 55,504). (SENSITIVITY ANALYSIS)

Diet group	*CO_2 (kg/d)*	*CH_4 (g/d)*	*N_2O (g/d)*
Vegans	2.02 (1.71, 2.73)	4.17 (2.99, 6.05)	0.67 (0.50, 0.95)
Vegetarians	3.33 (2.58, 4.46)	20.37 (15.91, 40.82)	0.99 (0.68, 1.44)
Fish-eaters	3.85 (3.04, 4.99)	23.01 (18.18, 44.45)	1.11 (0.77, 1.55)
Low meat-eaters	4.03 (3.13, 5.14)	27.74 (22.45, 50.42)	1.24 (0.91, 1.68)
Medium meat-eaters	5.40 (3.91, 7.36)	41.17 (33.10, 69.29)	1.76 (1.26, 2.38)
High meat-eaters	8.35 (5.64, 13.91)	74.68 (59.25, 129.01)	3.02 (2.04, 4.45)

All results are presented as median (2.5th percentile, 97.5th percentile) from a Monte Carlo analysis with 1000 iterations

Supplementary Table 6 Dietary greenhouse gas emissions by diet group aggregated using the 100-year Global Warming Potential (GWP100), 100-year Global Temperature change Potential (GTP100), and 20-year Global Warming Potential (GWP20), standardised by age and gender *but not standardised by kcal consumption*. Results presented for all adults (n = 55,504). (SENSITIVITY ANALYSIS)

Diet group	*GWP100 CO_2e (kg/d)*	*GTP100 CO_2e (kg/d)*	*GWP20 CO_2e (kg/d)*
Vegans	2.32 (1.97, 3.13)	2.27 (1.93, 3.07)	2.57 (2.16, 3.39)
Vegetarians	4.17 (3.32, 5.81)	3.85 (3.05, 5.22)	5.37 (4.39, 8.01)
Fish-eaters	4.80 (3.91, 6.35)	4.45 (3.59, 5.77)	6.17 (5.09, 8.90)
Low meat-eaters	5.13 (4.10, 6.70)	4.71 (3.72, 6.04)	6.77 (5.54, 9.51)
Medium meat-eaters	7.08 (5.33, 9.43)	6.42 (4.76, 8.52)	9.61 (7.39, 13.15)
High meat-eaters	11.69 (8.07, 18.05)	10.25 (7.08, 16.08)	16.84 (11.74, 25.61)

All results are presented as median (2.5th percentile, 97.5th percentile) from a Monte Carlo analysis with 1000 iterations

Supplementary Table 7 Land use, water use, eutrophication and biodiversity impact by diet group, standardised by age and gender *but not standardised by kcal consumption*. Results presented for all adults (n = 55,504). (SENSITIVITY ANALYSIS)

Diet group	*Land use (m^2/d)*	*Water use (m^3/d)*	*Eutrophication (gPO_4e/d)*	*Biodiversity impact (10^{-12} species extinction/d)*
Vegans	4.13 (3.39, 5.58)	0.39 (0.24, 0.72)	10.05 (8.11, 15.11)	1.06 (0.69, 2.38)
Vegetarians	6.06 (5.09, 9.40)	0.53 (0.38, 0.89)	17.34 (14.46, 22.05)	2.09 (1.20, 5.46)
Fish-eaters	6.44 (5.31, 9.85)	0.72 (0.49, 1.63)	21.39 (17.63, 26.81)	2.15 (1.26, 5.63)
Low meat-eaters	7.94 (5.72, 12.21)	0.68 (0.47, 1.61)	22.56 (18.39, 27.56)	2.21 (1.30, 5.76)
Medium meat-eaters	11.44 (7.52, 25.92)	0.79 (0.55, 2.01)	29.99 (24.34, 37.05)	2.83 (1.61, 6.96)
High meat-eaters	19.24 (11.89, 68.55)	1.02 (0.72, 2.33)	46.82 (35.99, 59.95)	4.28 (2.23, 10.14)

All results are presented as median (2.5th percentile, 97.5th percentile) from a Monte Carlo analysis with 1000 iterations

Supplementary Table 8 Relative environmental footprint from greenhouse gas emissions (CO_2, CH_4 and N_2O) of diet groups in comparison to high meat-eaters (>100g/d)

Diet group	*Ratio, median (2.5th percentile, 97.5th percentile)*		
	CO_2	*CH_4*	*N_2O*
Vegans	0.303 (0.170, 0.455)	0.065 (0.037, 0.097)	0.274 (0.167, 0.423)
Vegetarians	0.463 (0.262, 0.666)	0.320 (0.180, 0.495)	0.379 (0.226, 0.557)
Fish-eaters	0.532 (0.306, 0.743)	0.356 (0.199, 0.526)	0.418 (0.262, 0.604)
Low meat-eaters	0.572 (0.378, 0.749)	0.451 (0.302, 0.595)	0.491 (0.351, 0.648)
Medium meat-eaters	0.714 (0.564, 0.838)	0.629 (0.510, 0.746)	0.659 (0.549, 0.776)
High meat-eaters	1.000	1.000	1.000

Supplementary Table 9 Relative environmental footprint from aggregated greenhouse gas emissions (GWP100, GTP100 and GWP20) of diet groups in comparison to high meat-eaters (>100g/d)

Diet group	*Ratio, median (2.5th percentile, 97.5th percentile)*		
	GWP100	*GTP100*	*GWP20*
Vegans	0.251 (0.151, 0.370)	0.277 (0.164, 0.411)	0.194 (0.120, 0.283)
Vegetarians	0.416 (0.260, 0.603)	0.434 (0.261, 0.626)	0.391 (0.237, 0.554)
Fish-eaters	0.471 (0.298, 0.662)	0.492 (0.305, 0.694)	0.441 (0.268, 0.606)
Low meat-eaters	0.525 (0.378, 0.689)	0.541 (0.380, 0.712)	0.506 (0.353, 0.650)
Medium meat-eaters	0.681 (0.564, 0.797)	0.694 (0.567, 0.814)	0.661 (0.547, 0.774)
High meat-eaters	1.000	1.000	1.000

Supplementary Table 10 Relative environmental footprint from land use, water use, eutrophication potential and biodiversity impact of diet groups in comparison to high meat-eaters (>100g/d)

Diet group	*Ratio, median (2.5th percentile, 97.5th percentile)*			
	Land use	*Water use*	*Eutrophication*	*Biodiversity impact*
Vegans	0.251 (0.071, 0.445)	0.464 (0.210, 0.810)	0.270 (0.194, 0.405)	0.343 (0.120, 0.653)
Vegetarians	0.359 (0.101, 0.599)	0.611 (0.290, 0.836)	0.428 (0.327, 0.563)	0.648 (0.245, 1.023)
Fish-eaters	0.373 (0.106, 0.628)	0.805 (0.667, 0.980)	0.519 (0.396, 0.667)	0.660 (0.255, 0.972)
Low meat-eaters	0.438 (0.207, 0.654)	0.800 (0.689, 0.910)	0.574 (0.496, 0.684)	0.692 (0.336, 0.919)
Medium meat-eaters	0.610 (0.432, 0.767)	0.881 (0.798, 0.973)	0.724 (0.671, 0.801)	0.806 (0.526, 0.935)
High meat-eaters	1.000	1.000	1.000	1.000

Supplementary Table 11 Relative environmental footprint from greenhouse gas emissions (CO_2, CH_4 and N_2O) of diet groups in comparison to high meat-eaters (>100g/d), *not standardised by kcal consumption* (SENSITIVITY ANALYSIS).

Diet group	*Ratio, median (2.5th percentile, 97.5th percentile)*		
	CO_2	*CH_4*	*N_2O*
Vegans	0.248 (0.141, 0.371)	0.054 (0.031, 0.080)	0.225 (0.138, 0.345)
Vegetarians	0.405 (0.232, 0.578)	0.282 (0.160, 0.430)	0.332 (0.200, 0.486)
Fish-eaters	0.472 (0.274, 0.653)	0.317 (0.179, 0.463)	0.371 (0.235, 0.529)
Low meat-eaters	0.481 (0.317, 0.627)	0.377 (0.252, 0.497)	0.411 (0.293, 0.542)
Medium meat-eaters	0.632 (0.498, 0.741)	0.554 (0.448, 0.660)	0.581 (0.482, 0.684)
High meat-eaters	1.000	1.000	1.000

Supplementary Table 12 Relative environmental footprint from aggregated greenhouse gas emissions (GWP100, GTP100 and GWP20) of diet groups in comparison to high meat-eaters (>100g/d), *not standardised by kcal consumption* (SENSITIVITY ANALYSIS).

Diet group	*Ratio, median (2.5th percentile, 97.5th percentile)*		
	GWP100	*GTP100*	*GWP20*
Vegans	0.207 (0.125, 0.302)	0.228 (0.136, 0.333)	0.160 (0.099, 0.230)
Vegetarians	0.365 (0.229, 0.526)	0.381 (0.230, 0.543)	0.343 (0.210, 0.483)
Fish-eaters	0.418 (0.267, 0.584)	0.437 (0.272, 0.611)	0.391 (0.241, 0.537)
Low meat-eaters	0.441 (0.316, 0.578)	0.455 (0.319, 0.595)	0.424 (0.296, 0.545)
Medium meat-eaters	0.602 (0.497, 0.705)	0.614 (0.499, 0.720)	0.584 (0.482, 0.684)
High meat-eaters	1.000	1.000	1.000

Supplementary Table 13 Relative environmental footprint from land use, water use, eutrophication potential and biodiversity impact of diet groups in comparison to high meat-eaters (>100g/d), *not standardised by kcal consumption* (SENSITIVITY ANALYSIS).

Diet group	*Ratio, median (2.5th percentile, 97.5th percentile)*			
	Land use	*Water use*	*Eutrophication*	*Biodiversity impact*
Vegans	0.206 (0.059, 0.362)	0.383 (0.177, 0.655)	0.220 (0.160, 0.327)	0.280 (0.101, 0.530)
Vegetarians	0.314 (0.090, 0.524)	0.531 (0.255, 0.724)	0.374 (0.285, 0.491)	0.563 (0.219, 0.890)
Fish-eaters	0.331 (0.097, 0.554)	0.706 (0.588, 0.855)	0.458 (0.349, 0.587)	0.581 (0.229, 0.859)
Low meat-eaters	0.368 (0.172, 0.550)	0.669 (0.578, 0.762)	0.479 (0.413, 0.573)	0.580 (0.284, 0.769)
Medium meat-eaters	0.538 (0.377, 0.681)	0.778 (0.706, 0.856)	0.639 (0.591, 0.708)	0.714 (0.465, 0.830)
High meat-eaters	1.000	1.000	1.000	1.000
